# An optogenetic arrhythmia model to study catecholaminergic polymorphic ventricular tachycardia mutations

**DOI:** 10.1038/s41598-017-17819-8

**Published:** 2017-12-13

**Authors:** Elisabeth Fischer, Alexander Gottschalk, Christina Schüler

**Affiliations:** 1 Buchmann Institute for Molecular Life Sciences, Goethe University, Max von Laue Strasse 15, D-60438 Frankfurt, Germany; 2 Institute of Biophysical Chemistry, Goethe University, Max von Laue Strasse 15, D-60438 Frankfurt, Germany; 3 Cluster of Excellence Frankfurt – Macromolecular Complexes, Goethe University, Max von Laue Strasse 15, D-60438 Frankfurt, Germany; 4 Present Address: University of Edinburgh, Centre for Integrative Physiology, Hugh Robson Building, George Square, Edinburgh, EH8 9XE UK

## Abstract

Catecholaminergic polymorphic ventricular tachycardia (CPVT) is a condition of abnormal heart rhythm (arrhythmia), induced by physical activity or stress. Mutations in ryanodine receptor 2 (RyR2), a Ca^2+^ release channel located in the sarcoplasmic reticulum (SR), or calsequestrin 2 (CASQ2), a SR Ca^2+^ binding protein, are linked to CPVT. For specific drug development and to study distinct arrhythmias, simple models are required to implement and analyze such mutations. Here, we introduced CPVT inducing mutations into the pharynx of *Caenorhabditis elegans*, which we previously established as an optogenetically paced heart model. By electrophysiology and video-microscopy, we characterized mutations in *csq-1* (CASQ2 homologue) and *unc-68* (RyR2 homologue). *csq-1* deletion impaired pharynx function and caused missed pumps during 3.7 Hz pacing. Deletion mutants of *unc-68*, and in particular the point mutant UNC-68(R4743C), analogous to the established human CPVT mutant RyR2(R4497C), were unable to follow 3.7 Hz pacing, with progressive defects during long stimulus trains. The pharynx either locked in pumping at half the pacing frequency or stopped pumping altogether, possibly due to UNC-68 leakiness and/or malfunctional SR Ca^2+^ homeostasis. Last, we could reverse this ‘worm arrhythmia’ by the benzothiazepine S107, establishing the nematode pharynx for studying specific CPVT mutations and for drug screening.

## Introduction

CPVT is an inherited disturbance of the heart rhythm (arrhythmia) induced by adrenergic stress. It derives from an alteration of intracellular Ca^2+^ handling, involving the Ca^2+^-induced Ca^2+^ release (CICR) mechanism of myocytes. If untreated, CPVT is highly lethal. The exact prevalence of CPVT is not known, but it has been estimated to be around 1:10,000^1^. The mean age of symptom onset is between 7 and 8 years^2^, but a wide range of ages has been observed. Most mutations that have been linked to CPVT are found in two genes, ryanodine receptor 2 (RyR2; 50–55%) and calsequestrin 2 (CASQ2; 3–5%), two proteins fundamentally involved in regulation of intracellular Ca^2+^ in cardiac myocytes^3,4^. The cardiac RyR2 is a Ca^2+^ release channel located in the SR. During CICR, Ca^2+^ is provided by the activated L-type voltage-gated Ca^2+^ channel (VGCC; Ca_v_1.2) and binds to RyR2 thus triggering the opening of the channel, allowing fast Ca^2+^ efflux from the SR and subsequent muscle contraction. After reuptake into the SR lumen by the SERCA complex (Sarcoplasmic/Endoplasmic Reticulum Calcium transporting ATPase), Ca^2+^ is stored there at high concentrations. Calsequestrin is the major Ca^2^^+^ sequestering protein in the SR. It forms higher-order dynamic structures of monomers, dimers or long chains, depending on the Ca^2+^ concentration. Polymerization of calsequestrin occurs by back-to-back (C-terminal) and front-to-front (N-terminal) interactions^5,6^. In mammals calsequestrin is linked to the RyR through an interaction with triadin^7^ and junctin^8^.

For calsequestrin there is only one isoform present in *C. elegans*, CSQ-1, which shows ~40% sequence identity and 50–60% similarity to the human CASQ2. Cho *et al*.^9^ reported that UNC-68 appeared to affect the correct localization of CSQ-1 *in vivo*. Based on the genome sequence, there are no obvious homologs of junctin and triadin in *C. elegans*, therefore a direct interaction of the positively charged CSQ-1 C-terminus with the negatively charged luminal loops of UNC-68 is postulated^10^.

In vertebrates three RyR isoforms are present, whereas *C. elegans* has a single RyR gene encoded by the *unc-68* locus^11^. The UNC-68 protein of *C. elegans* shares 45% sequence identity and 63% homology with the human cardiac RyR2. A low-affinity Ca^2+^ binding site specific for RyR1 (aa 1872-1923) is not present in UNC-68, arguing that it is more related to RyR2. UNC-68 is expressed in the terminal bulb and posterior isthmus muscle cells of the pharynx^11^, but also in body wall muscles and neurons. *unc-68* mutants are viable, but were identified as being defective in locomotion^12^ and to exhibit defects in pharyngeal pumping. In addition to visible defects in muscle function, *unc-68* mutants grow more slowly and have fewer offspring than the wild type. In contrast, mutant mice lacking RyR2 die as embryos with morphological abnormalities in the heart tube^13^.

Up to now, many different (>170) CPVT mutations and polymorphisms are known^14^ (http://triad.fsm.it/cardmoc/). These result in a Ca^2+^ leakage from the SR, which leads to cytosolic Ca^2+^ overload generating delayed afterdepolarizations (DADs) (by driving the electrogenic sodium-calcium exchanger, NCX), triggered activity, and ventricular arrhythmias, in particular under adrenergic conditions^15^. Disease-causing mutations have also been identified in RyR1, and are linked to malignant hyperthermia or central core disease^16–19^. Recently, the significance of RyR function for aging was shown by single amino acid modifications, which conferred a reduction in lifespan and an accelerated decline in muscle integrity with age in *C. elegans* ^20^.

In this study we aimed to model CPVT arrhythmias in the *C. elegans* pharynx, a rhythmically active muscular pump that we previously established as an optogenetically controlled arrhythmia test system^21^. We thus examined two available mutations of *C. elegans* calsequestrin. First, we analyzed possible defects of a deletion mutant. In addition, we tested a mutation at position P319 (mutated to S), a residue that corresponds to P308 in human CASQ2, and mutation of which is an established cause of CPVT. This mutation (however, to L, not to S) caused decreased Ca^2+^-selectivity during polymerization of purified CASQ2-P308L protein^5,22,23^ as a structural disruptor for α-helices and as a turning point in β-sheets, thus it was suggested that a substitution of P308 causes a profoundly altered conformation of the CASQ2 protein, and that this could lead to reduced Ca^2+^ binding^22^. Therefore, we expected that any alteration of this proline in *C. elegans* CSQ-1 would also cause problems in folding or conformation, and consequently in Ca^2+^ binding capacity. Even though the worm allele introduces a polar residue instead of an aliphatic one, the structural consequences could be equally strong.

In the case of RyR2, we inserted two mutations (R2474S and R4497C) in *C. elegans* UNC-68, identified in human patients with CPVT^24^, and demonstrated in mouse models to be causative to CPVT^25,26^. They represent two of the most investigated CPVT mutations in animal models or cell systems. Nevertheless, the mechanisms by which they induce arrhythmia are still not completely resolved. R2474S resides in the helical domain 1 segment 6b^27^. One study suggested that the CPVT-associated RyR2 mutation results in leaky RyR2 channels due to decreased binding of calstabin-2 (FKBP12.6), which stabilizes the closed state of the channel^25^. However, more recently it was found that the RyR2-FKBP12.6 interaction was not disrupted in R2474S/ + knock-in mice. These authors suggested that the R2474S mutation in the central domain of RyR2 induced a defective interaction between the central and the N-terminal domains, thus causing channel dysfunction^28^. Jiang *et al*.^29^ could also not observe effects of CPVT mutations on FKBP12.6 binding. The mutation R4497C is located in the S0 segment of the RyR channel domain^27^ affecting the channel gating and creating a SR Ca^2+^ leak^30^. A decreased binding of FKBP12.6 was also detected for this mutation^25^. However, in other studies a normal RyR2-FKBP12.6 interaction was shown in RyR2^R4496C+/−^ mice^31^ or in coimmunoprecipitation studies from resting HL-1 cardiomyocytes (CM)^32^. The reasons for these discrepancies are unclear.

To enable drug development for treatment of specific arrhythmias, but also to study distinct arrhythmia types, simple model systems are required, that allow implementing patient-specific mutations. Up to now, only a handful of mouse models for CPVT exist^25,26,33–37^. Few RyR2 mutations have already been characterized in CM derived from induced pluripotent stem cells (iPSCs) and reflect basic aspects of CPVT that can be assessed well on the single-cell level^38–45^. Novak *et al*.^46^ found in iPSC-CMs with the D307H CPVT mutation of calsequestrin that the β-adrenergic agonist isoproterenol causes DADs, oscillatory arrhythmic prepotentials, after-contractions and a diastolic [Ca^2+^]_i_ rise. However, iPSCs are an expensive and complicated experimental system, precluding large scale screening approaches, and, more importantly, they cannot reflect the complex disease as it is present in an intact organ. For this reason we assessed the pharynx, the feeding organ of *C. elegans*, which is a rhythmically active muscular pump^47^, as a heart model^21^. Beside the easily accessible genetics and its high throughput potential, there are observations suggesting that the pharynx is orthologous to the vertebrate heart^48^. Both the pharynx and the heart are tubes transporting material along their lumina. The pharynx utilizes homologues of most of the ion channels, pumps and transporters defining human CM physiology. Similar to the mammalian heart, the pharynx muscle cells are connected by gap junctions^49^. The *C. elegans* EGL-19 L-type voltage dependent Ca^2+^ channel α1-subunit maintains depolarization during the plateau phase and sustains muscle contraction similar to the cardiac action potential (AP). In fact, with a plateau phase of ~200 ms, the pharynx AP resembles the shape of the human cardiac AP better than that of the mouse. EGL-19 slowly inactivates, and membrane repolarization is executed by a voltage-gated K^+^-channel, encoded by *exp-2* ^50^ which, though homology is low, is functionally similar to the human ether-a-go-go-related gene (hERG) channel.

Spontaneous pharynx pumping is too irregular to allow arrhythmia detection. To yield millisecond-precise and stable rhythmicity, we optically paced the pharynx using channelrhodopsin-2 (ChR2)^21^. ChR2(H134R), a gain-of-function variant, was directed to the plasma membrane of the pharyngeal muscle cells by the specific promoter *pmyo-2* and its expression allows stimulation of pumping. We assessed pharynx pumping by extracellular recordings (electropharyngeograms, EPGs), similar to electrocardiograms, of dissected (cut-head preparation) or intact worms, which allows accurate measurements of pump rate and duration, and distinguishing muscle activity of different parts of the pharynx, as well as activity of pharyngeal neurons^51,52^. Corpus and terminal bulb contraction/excitation, as well as their relaxation/repolarization, can be automatically analyzed^53^. In addition, we used a video-microscopy based method, which allows recording of multiple intact animals simultaneously^21^. Importantly, ChR2-paced pumping of the pharynx is not influenced by neuronal input^21^.

Here, we extended our pharynx arrhythmia model to CPVT like arrhythmias. We characterized the role of RyR and calsequestrin in deletion mutants. Further we introduced different CPVT-related mutations of *csq-1* and *unc-68* and examined their effect on optogenetically paced pumping, exhibiting clear arrhythmia phenotypes and progressive loss of the ability to lock into the pacing. Finally, we demonstrate ameliorating effects of the benzothiazepine S107, emphasizing that our model can be used to identify and/or test allele-specific drugs.

## Results

### Deletion of calsequestrin causes arrhythmia in the *C. elegans* pharynx

**Figure 1:**
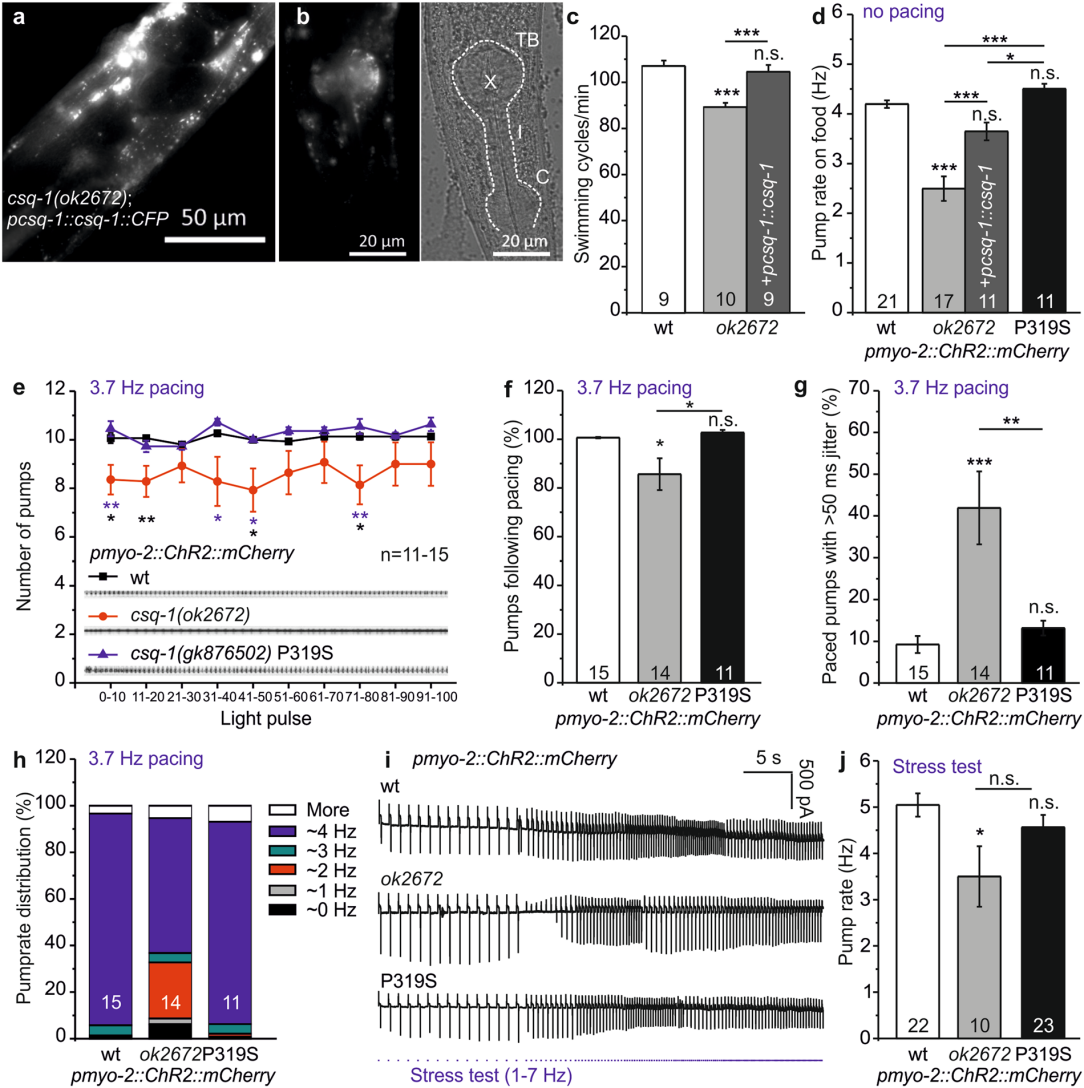
CSQ-1 deletion and the point mutation P319S affect pharyngeal pumping. (**a**) Expression of *pcsq-1::csq-1::CFP* in BWM cells and (**b**) pharynx muscle of the deletion mutant *csq-1(ok2672)*. Scale bars: 50 μm and 20 μm, as indicated. Left panel: Fluorescence micrograph, right panel: DIC image; dashed lines indicate pharynx position. Structural features of the pharynx are labeled: TB (terminal bulb), I (isthmus), C (corpus), X (grinder). (**c**) Swimming cycles/min and (**d**) pump rate on food, of *csq-1(ok2672)* deletion mutants, as well as full length *pcsq-1::csq-1::CFP* rescue in *pmyo-2::ChR2::mCherry* background. Also analyzed (**d**) is the point mutation CSQ-1(P319S), compared to wt (number of animals tested is indicated at the base of each bar). (**e**) Number of pumps evoked at 3.7 Hz pulse frequency was counted for 100 light stimuli and averaged in 10 stimuli bins for the indicated number of animals. Original kymographs are depicted for wt (black), *csq-1(ok2672)* (red) and *csq-1(gk876502)* (blue; P319S). (**f**) Percentage of light stimuli inducing a pump across 100 light stimuli (data in e). (**g**) Percentage of pumps that were induced with a deviation of > 50 ms following a light stimulus (here defined as ‘jitter’). Shown (f and g) are wt (white), *csq-1(ok2672)* deletion mutant (grey) and P319S mutant (black). (**h**) Pump rate distribution (%) at 3.7 Hz pacing (white: > 4.5 Hz, blue: 3.3–4.5 Hz, green: 2.5–3.3 Hz, red: 1.6–2.5 Hz, grey: 0.5–1.6 Hz, black: 0 Hz) in the indicated *csq-1* mutants, compared to wild type (wt) (n = 11–15). (**i**) Original EPG recordings of cut-head preparations and (**j**) mean of maximal pump rate of deletion and P319S mutant achieved in a stress test (470 nm, 10 ms pulses at stepwise increasing pulse rate (1 Hz steps, 5 s each step, 1–7 Hz), as indicated by blue tick marks) compared to wt (n = 10–23). Statistically significant differences, 1-way ANOVA and Bonferroni post-hoc test: ***p < 0.001; **p < 0.01; *p < 0.05.

To demonstrate its function in the pharynx, we expressed CSQ-1, fused to CFP at its C-terminus, under its own promoter in *csq-1* deletion mutants (Fig. 1a,b). Expression was observed in body wall muscle (BWM) cells and in the muscles of the pharyngeal terminal bulb. CSQ-1::CFP showed aggregated expression, that did not resemble the punctuate and mesh-like pattern as previously observed in antibody-staining for CSQ-1^10^. One reason could be overexpression of CSQ-1, or a disturbed back-to-back dimerization, due to an interference of the C-terminal CFP fusion. Nonetheless, we achieved complete rescue of *csq-1(ok2672)* deletion mutant phenotypes in a swimming locomotion assay depending on normal BWM function (Fig. 1c). Likewise, *csq-1(ok2672)* led to reduced pumping ability in the presence of bacterial food, and a complete rescue was observed by CSQ-1::CFP expression (Fig. 1d), while the point mutation P319S did not show significant differences to WT. The *csq-1(ok2672)* deletion mutant was unable to follow the 3.7 Hz optogenetic pacing (p ≤ 0.05; kymographic video analyses; Fig. 1e,f). In addition, the pump frequency of the deletion mutant showed an increased ‘jitter’ (here, defined as a deviation of more than 50 ms from the timing that would be achieved at precisely 3.7 Hz frequency, as dictated by the light pulses; Fig. 1g), resulting in periods with reduced pump frequency of about 2 Hz (Fig. 1h) and a reduced maximal pump rate in a stress test with a stepwise increase of the stimulation frequency (1 Hz steps, 5 s each step, 1–7 Hz) via EPG recordings of cut-head preparations (Fig. 1i,j). In humans, absence of CASQ2, induced by nonsense mutations like R33X, causes severe forms of CPVT^54^. The observed phenotypes in the *C. elegans* pharynx could thus be considered an analogous form of ‘worm arrhythmia’. Against our expectations, the point mutation CSQ-1(P319S) did not show significant defects. Probably the polar serine residue is not affecting the Ca^2+^ binding and polymerization properties as much as the aliphatic leucine found in the human P308L mutation^23^ (Fig. 1d–j). There was no effect of the *csq-1* mutations on pump duration (Supplementary Fig. 1a,b), arguing that the phenotypes were not induced by prolonged AP duration, as for our earlier pharynx model of Timothy syndrome (long QT8; LQT8)^21^.

### Absence of the RyR/UNC-68 affects pharynx pumping

**Figure 2:**
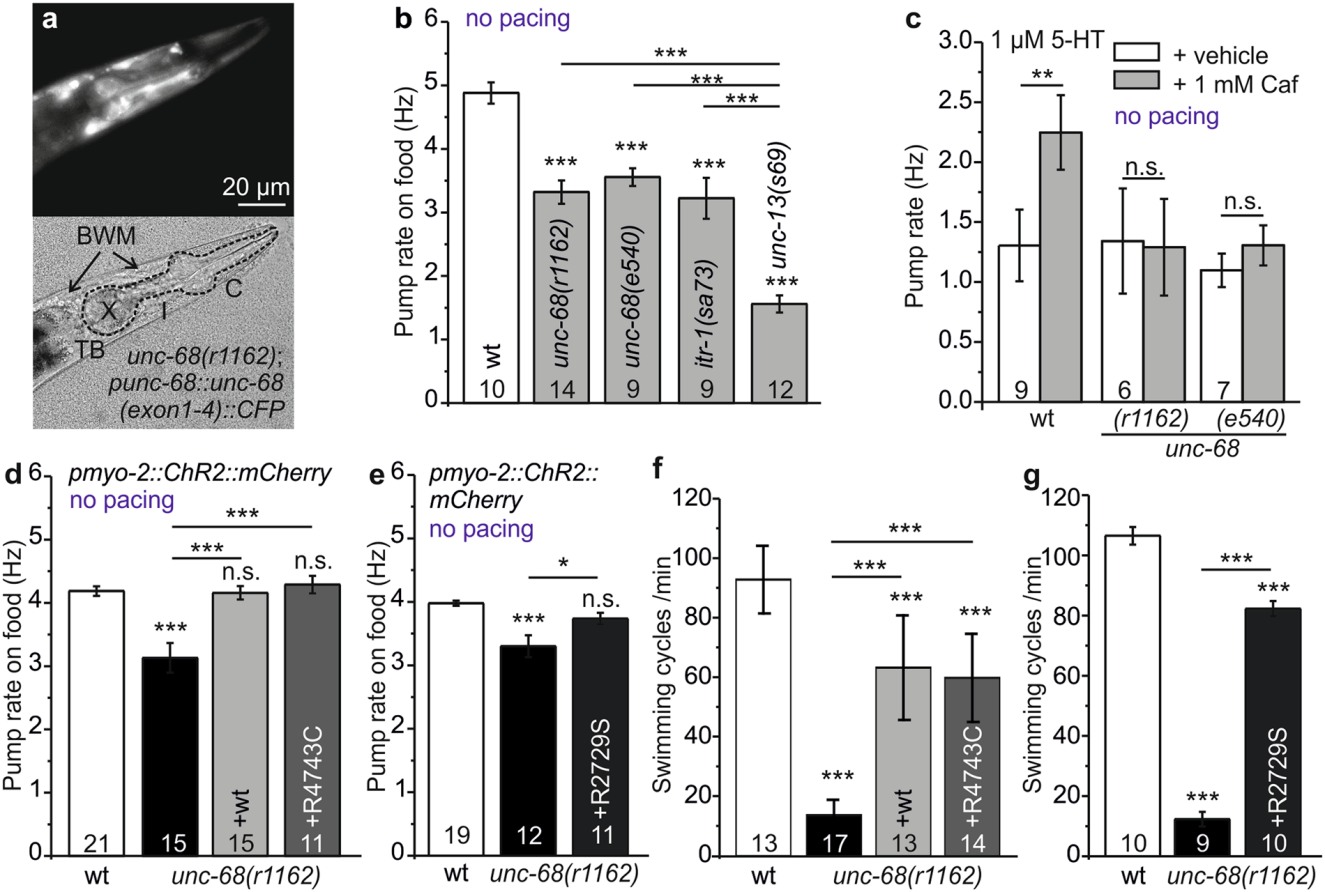
Introduction of CPVT point mutations R4743C and R2729S into the *C. elegans* RyR2 (UNC-68). (**a**) Expression of *punc-68::unc-68(exon1–4)::CFP* in pharynx and BWM of deletion mutant *unc-68(r1162)*. Dashed lines indicate pharynx position and features, as in Fig. 1a, arrows indicate BWM cells. (**b**) Pump rate on food of *unc-68(r1162)* and *unc-68(e540)* deletion mutants, compared to wt, IP3 receptor (*itr-1(s73)*) and UNC-13 synaptic vesicle priming factor (*unc-13(s69)*) reduction of function mutants (n = 9–14). (**c**) Stimulation of pumping by 1 µM serotonin (5-HT), before (white bars) and after activation of wt UNC-68 by application of 1 mM caffeine (grey bars). Shown is the pump rate (Hz) deduced from EPG recordings of cut-head preparations compared to *unc-68* deletion mutants *r1162* and *e540* (n = 6–9; paired t-Test). (**d**,**e**) Pump rate on food (Hz) of *unc-68(r1162)* deletion mutant, its rescue with a fosmid encoding the wt genomic *unc-68* locus as well as the R4743C (n = 11–21) and (e) R2729S point mutant engineered fosmids, compared to wt (n = 11–19). (**f,g**) Mean and SEM of swimming cycles/min of deletion mutant *unc-68(r1162)*, rescue with wt fosmid and the R4743C point mutant fosmid, as well as the R2729S point mutant fosmid (**g**) compared to wt (number of animals as indicated). Statistically significant differences, 1-way ANOVA and Bonferroni post-hoc test: ***p < 0.001; **p < 0.01; *p < 0.05.

To modify the *C. elegans* RyR (UNC-68), we used fosmid recombineering. The fosmid we used contains a slightly truncated promoter, thus we first verified if this promoter fragment is expressed in pharyngeal muscles. This was the case, i.e. the *punc-68* promoter fragment included in the fosmid, transcriptionally fused to CFP, was expressed in pharyngeal muscles and BWMs (Fig. 2a). *unc-68* mutants were identified as showing uncoordinated locomotion^12^, and to exhibit defects in pharyngeal pumping. In addition, *unc-68* mutants grow slowly and have fewer offspring than wild type (wt). The *unc-68(r1162)* mutant eliminates a large part of the channel pore domain, thus *r1162* can be considered a molecular ‘null’ allele^55^.

Behavioral and morphological phenotypes of *unc-68* mutant animals suggest that intracellular Ca^2+^-release is not essential for excitation-contraction (EC) coupling in *C. elegans*, but may rather act to amplify a plasma membrane mediated calcium influx that itself is sufficient for contraction^11,55^. Thus, we observed a reduction of spontaneous pump rate on food of only 32% in the *unc-68(r1162)* mutant compared to wt (Fig. 2b). A second deletion mutant, *unc-68(e540)*, and a mild reduction of function mutant of the inositol trisphosphate (IP_3_)-receptor *itr-1(sa73)* exhibited a similarly reduced spontaneous pump rate (in the presence of food). A deletion of the presumptive FKBP12.6 homolog *fkb-2* (*fkb-2(ok3007);* Supplementary Fig. 2a) led to an even weaker reduction of spontaneous pump rate on food (15%; Supplementary Fig. 2b) than did *unc-68* deletions. Interestingly, for spontaneous pumping, eliminating synaptic transmission in the *unc-13(s69)* mutant^56^ caused a more severe phenotype than *unc-68* deletions (Fig. 2b), i.e. slower pump rate, demonstrating a strong neuronal influence on food-stimulated pumping (at high rates). Paced pumping, however, was not affected by *unc-13(s69)* (Supplementary Fig. 3).

To demonstrate the conservation of function of the RyR between humans and *C. elegans*, we incubated animals with caffeine (1 mM), a well-known activator of RyR. In EPG recordings, a 72% increase of the pump rate was observed in wt (Fig. 2c; note that spontaneous pumping under EPG conditions occurs at a low rate resembling pumping in the absence of food), indicating an activation of UNC-68 by caffeine, while no effect of caffeine was observed in two different mutants lacking UNC-68 (*r1162* and *e540*), and further suggesting an important contribution of the RyR to caffeine induced pumping.

### *unc-68* transgenes bearing homologous CPVT-mutations rescue *unc-68* deletion mutant phenotypes

Since UNC-68 affects pharynx function, we wanted to assess if point mutations known to affect RyR2 and cardiac function in humans would also affect pumping. Because *unc-68* is a very large gene (the genomic sequence is 27 kb, the cDNA is 15 kb), we introduced mutations into a rescuing fosmid containing *unc-68* by recombineering. We chose two mutations (described above) from human CPVT patients that were demonstrated to cause arrhythmia also in mice: The R4743C mutation, analogous to the CPVT mutation R4497C in humans (R4496C in mice; resulting in a leaky channel^24,30^), and R2729S analogous to the CPVT mutation R2474S in humans and mice (resulting in weak calstabin-2 binding^24,25^ and/or defective interdomain interactions^28^). The engineered fosmids were used to generate transgenic lines containing extrachromosomal arrays, in the *unc-68(r1162)* mutant background. As a control, we also generated a rescuing transgene from the unmodified fosmid. Expression of mutant UNC-68 proteins, in the absence of the endogenous protein, resulted in complete (R4743C, Fig. 2d; R2729S, Fig. 2e) rescue of pumping behavior. The wt rescue line exhibited a complete rescue as well (Fig. 2d). The ryanodine receptor point mutations, which could be expected to cause a basal increase of cytosolic Ca^2+^, did not increase the spontaneous pump rate. This may be due to adaptation of the pharynx to this situation, or a potentially increased pump rate due to higher basal Ca^2+^ might be counteracted by stronger muscle contraction that is less readily ´released´ in time for the next pump. Swimming assays, probing UNC-68 function in BWMs, exhibited a partial rescue for both transgenes (Fig. 2f,g). The same was found for the wt fosmid. Thus, incomplete rescue of locomotion may be due to mosaicisms of the array expression, or an incomplete expression pattern of the fosmid.

### The R4743C mutation of RyR/UNC-68 causes arrhythmic phenotypes in the pharynx

**Figure 3:**
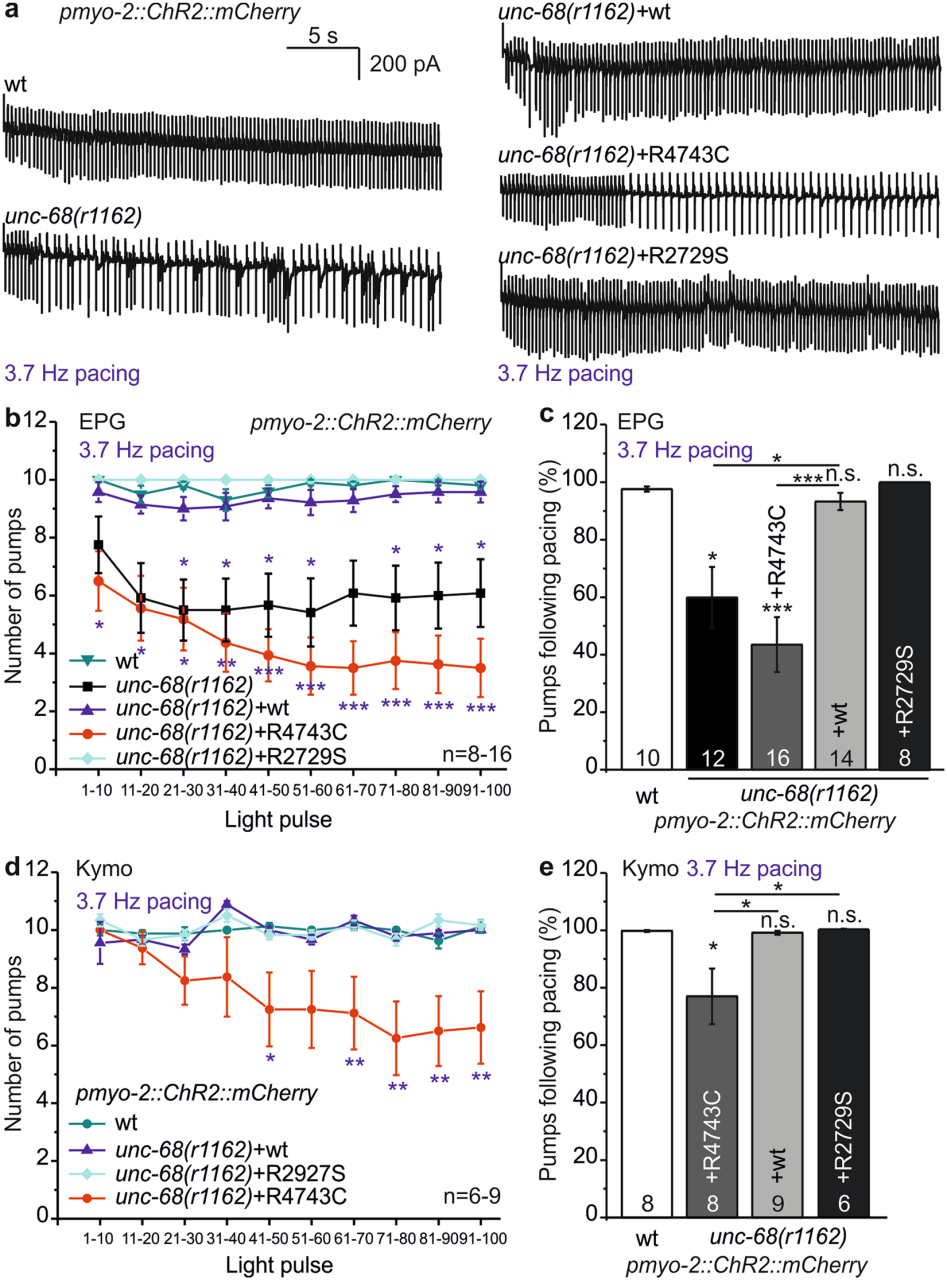
The R4743C mutation, analogous to human CPVT mutation R4497C, induces arrhythmia in the pharynx. (**a**) Original EPG recordings of intact, *pmyo-2::ChR2::mCherry-*expressing animals containing *unc-68* deletion mutant *r1162*, its rescue with wt *unc-68* fosmid, the R4743C or R2729S mutant fosmids, and wt. (**b**) Mean and SEM number of pumps achieved over 100 consecutive light stimuli (3.7 Hz, 35 ms), binned per 10 light pulses, as in Fig. 1e, but obtained by EPG recordings. Genotypes of the animals tested are indicated. (**c**) Percentage of successful light pulses followed by a pump, across all 100 light stimuli, as recorded in (b). (**d**,**e**) Analyses analogous to (**b**) and (**c**), but obtained by kymographic analysis of video recordings in intact *pmyo-2::ChR2::mCherry-*expressing animals. Statistically significant differences, 1-way ANOVA and Bonferroni post-hoc test: ***p < 0.001; **p < 0.01; *p < 0.05.

Next, we probed rhythmicity of pumping in the engineered CPVT pharynx models by optogenetic pacing. We presented 100 light pulses at a rate of 3.7 Hz and recorded pharynx activity by EPG-recordings of intact animals. Wild type animals and *unc-68(r1162)* mutants with the wt fosmid rescue could follow the pace rhythm faithfully until the end of the stimulus train (Fig. 3a). However, the *unc-68(r1162)* deletion mutant, as well as the mutant rescued with UNC-68(R4743C) exhibited pumping defects. To analyze these in a quantitative manner, we counted the actual pumps for each 10 light pulses. Both the deletion mutant as well as the point mutation R4743C could briefly follow the 3.7 Hz pumping period in the beginning of the stimulation protocol, but then exhibited a decrease of the pump rate, that was particularly pronounced for the R4743C CPVT mutant (Fig. 3b,c). This was also observed in kymographic video-analysis, in intact animals. The second CPVT-related mutation R2729S did not exhibit an inability to follow fast pacing (Fig. 3a–e). None of the *unc-68* mutations had an effect on pump duration (Supplementary Fig. 1c–e), thus, as for *csq-1*, excluding prolonged AP duration as a cause for the observed inability to lock into the pacing stimulus.

### The 1,4-benzothiazepine derivative S107 reverses the pump inability evoked by the CPVT mutation R4743C

Up to now β-blockers are the first therapeutic option for patients with CPVT. Although β-blockers reduce the occurrence of ventricular tachycardia, 30% of patients still experience cardiac arrhythmias and eventually require cardioverter defibrillator implantation to prevent cardiac arrest^1,3^. Thus, there is a strong motivation to identify more specific drugs to treat CPVT arrhythmias, particularly to enable treatment with respect to the various mutations affecting the different domains of RyR2 or CASQ2, and their interactions with other proteins. Ideally, mutation-(patient-)specific drugs may be identified that could prevent cardiac arrhythmias. The 1,4-benzothiazepine derivative K201 (a.k.a. JTV519) is an anti-arrhythmic drug currently under clinical investigation. However, K201 is a non-specific blocker of sodium, potassium and calcium channels^57^. Thus, there are’mixed‘ results for K201, with side-effects outweighing benefits^58^. Treatment with K201 enhanced the affinity of FKBP12.6 for RyR2, which stabilized the closed state of RyR2 and prevented the Ca^2+^ leak triggering arrhythmias^59^. Furthermore, K201 was effective in reducing SR Ca^2+^ leak by specifically regulating RyR2 opening at diastolic [Ca^2+^]_i_ in the absence of increased RyR2 phosphorylation at Ser2814, thus extending the potential use of K201 to conditions of acute cellular Ca^2+^ overload^60^. However, in another study on isolated myocytes, K201 treatment did not elicit a decrease in delayed after depolarizations and K201 failed to prevent induction of CPVT in RyR2 R4496C knock-in mice^31^.

**Figure 4:**
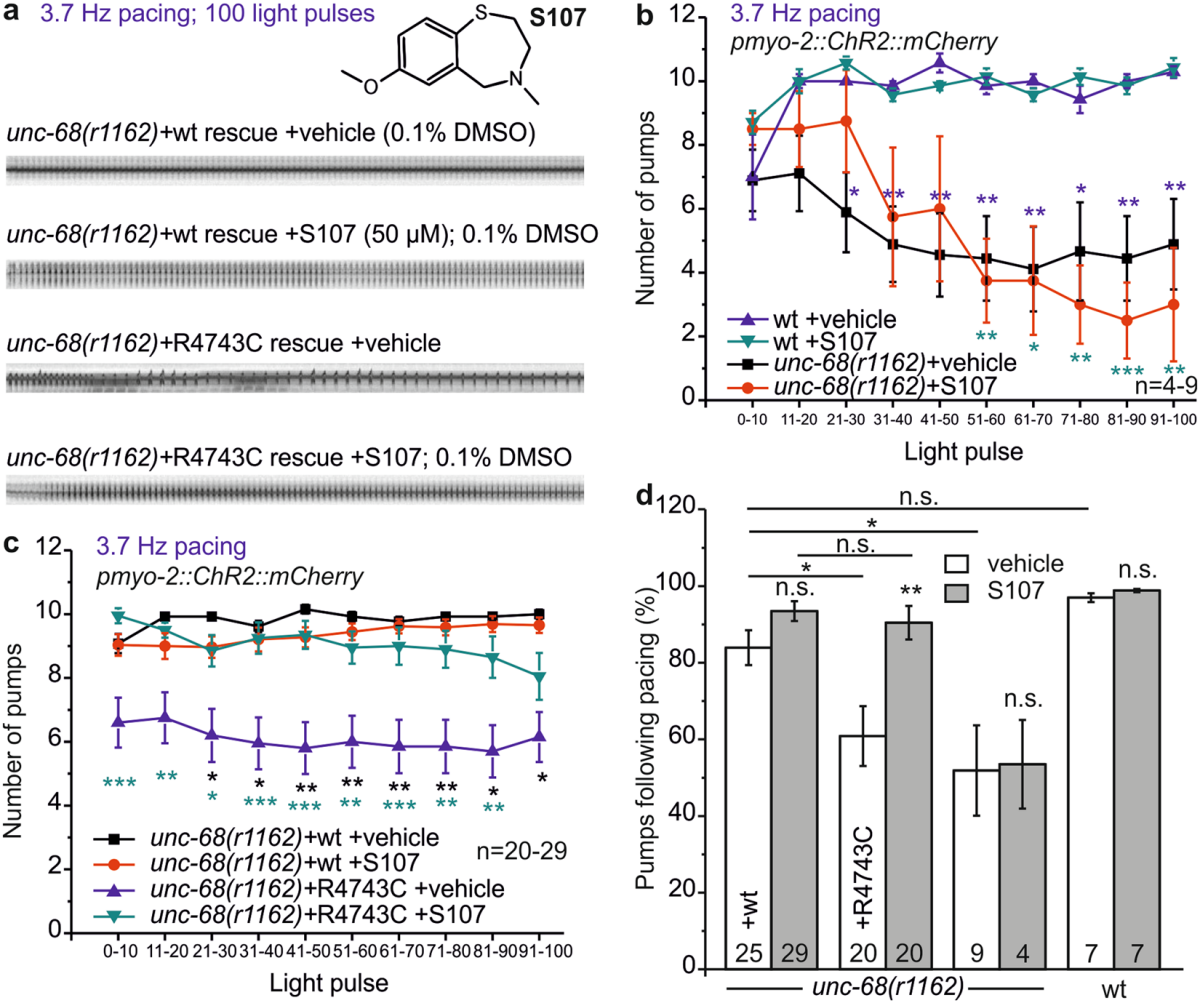
The benzothiazepine S107 reverses arrhythmia in the UNC-68(R4743C) mutant. (**a**) Original kymographs of video-microscopic recordings of the *unc-68(r1162)* rescue with wt and R4743C mutant fosmids, with or without incubation in 50 µM of the benzothiazepine S107 (inset) in 0.1% DMSO. (**b**) Mean and SEM number of pumps achieved over 100 consecutive light stimuli (3.7 Hz, 35 ms), binned per 10 light pulses, obtained by kymographic video analysis of intact, *pmyo-2::ChR2::mCherry-*expressing animals. As indicated, *unc-68(r1162)* deletion mutants, after 30 min incubation in 50 µM S107 (red) or without S107 (0.1% DMSO; black), were compared to wt with (green) or without (blue) S107 incubation (n = 4–9). (**c**) Analysis as in (**b**), but for *r1162* deletion mutants expressing the R4743C fosmid, with S107 (green) or without (blue), or the rescue wt fosmid (red and black, respectively) (n = 20–29). (**d**) Percentage of successful light pulses followed by a pump, across all 100 light stimuli, as recorded in (**b**) and (**c**). Statistically significant differences, 1-way ANOVA and Bonferroni post-hoc test: ***p < 0.001; **p < 0.01; *p < 0.05.

Thus, we decided to test a novel RyR2-specific compound (S107), a more specific 1,4-benzothiazepine derivative, which in the rodent model enhanced the binding of FKBP12.6 to the mutant RyR2(R2474S) channel, inhibited the channel leak and prevented cardiac arrhythmias^25^. Sasaki *et al*.^43^ could recently show in iPSCs for another C-terminal CPVT mutation (I4587V) that the development of DADs in the presence of isoproterenol was significantly suppressed by S107. But, to our knowledge, S107 was never tested on R4497C (R4743C in *C. elegans*). In fact we found after a 30 min incubation in S107 (50 µM) a complete rescue of the pump ability of R4743C mutation bearing nematodes (Fig. 4a,c,d). Whereas kymographs showed that the R4743C mutants solely incubated in the vehicle DMSO (0.1%) quickly switched to pumping at half the pacing frequency, or sometimes stopped pumping at all, incubation in S107 enabled faithful 3.7 Hz pacing. In addition, there was no rescue of pump ability in the deletion mutant (Fig. 4b,d) indicating an *unc-68* (RyR) specific effect of S107, that was not elicited *via* other, unknown channels.

## Discussion

In a former study^21^ we established the *C. elegans* pharynx as a model for analyzing LQT8-like arrhythmogenic mutations affecting the Ca_v_1.2 VGCC. Optogenetic stimulation of pharynx muscle *via* ChR2 enables pacing at about 4 Hz (and up to 6 Hz) for > 1 minute. Here, we extended this model for CPVT-related mutations and characterized the role of RyR2- and CASQ2-mutations in *C. elegans* pumping, including homologous mutations causing CPVT in humans. The UNC-68(R4743C) mutation, homologous to human RyR2(R4497C), causing channel leakiness, provoked a progressive drop in the ability to follow pacing. The experimental drug S107, a derivative of 1,4-benzothiazepine, caused a complete reversion of the phenotype, emphasizing that the *C. elegans* pharynx can indeed be used as a simple, genetically amenable model for cardiac arrhythmias, and possibly also for drug screening.

Eliminating the Ca^2^^+^ sequestering protein CSQ-1 (allele *ok2672*) weakly affected swimming locomotion (16% reduced swimming cycles), but had a more pronounced effect on spontaneous pharynx pump rate on food (38% reduction), which could be reversed by over-expression of CSQ-1::CFP. Inconsistently, in previous work, no significant effect on *C. elegans* locomotion speed could be observed, neither by RNAi against *csq-1* nor in another deletion mutant *csq-1(jh109)* ^9,10^. Interestingly, CASQ2-null mice are viable and display normal SR Ca^2+^ release and contractile function under basal conditions, although they exhibit striking increases in SR volume^33^. Nevertheless, these mice exhibit severe forms of CPVT, analogous to the phenotypes observed in humans lacking CASQ2^54^.

Here, we observed an arrhythmic phenotype for the deletion mutant of *csq-1* in the paced pharynx model, which was unable to follow the 3.7 Hz stimulation (Fig. 1e) and in addition reached only reduced pump rates in the stress test (Fig. 1i,j). The point mutation P319S, similar to the human CPVT mutation P308L^22^, showed no significant deviation from pulse frequency. Either, exchange of proline for a polar serine residue in the *C. elegans* mutant P319S has less impact on the folding or conformational properties of calsequestrin than the aliphatic leucine in the human P308L mutation^23^. Alternatively, as found for the human D307H mutation (D318H in *C. elegans*), loss of function was related to the observed loss of normal binding of CASQ2(D307H) to junctin and triadin^61^. The fact that the *C. elegans* genome apparently does not encode triadin and junctin^10^ could explain why we did not observe any strong effect of the P319S mutation.

Homozygous deletions of the RyR2 gene are embryonically lethal in mice^13,62^. The *C. elegans* RyR is required for normal locomotion and pumping. However, the *unc-68* deletion mutant phenotype shows that UNC-68 is not essential for EC coupling or survival in the nematode. RyR could cooperate with the IP_3_ receptor for pumping at high rates, since a mild reduction of function mutant of *itr-1(sa73)* ^63^ shows a similarly reduced pump rate on food as *unc-68* deletions. Animals with strong *itr-1* loss-of-function alleles are sterile^64^.

We introduced the CPVT-related mutation R2729S into UNC-68 (R2474S in humans), but observed no effect on paced pump ability, only swimming locomotion was affected. Possibly, an interaction of UNC-68 with the *C. elegans* FKBP12.6 may be different than in vertebrates; this will have to be assessed in future work. A clear FKBP12.6 homolog is yet to be identified in *C. elegans*, which contains 8 members of the *fkb* gene class (we tested the presumably best homologue, FKB-2; Supplementary Fig. 2). Alternatively, the analogous *C. elegans* mutation R2729S does not impair the interaction between the two structural domains in UNC-68 (N-terminal and central domains) to the same extent as in human RYR2^28^. The *C. elegans* UNC-68 protein could have a slightly altered structure, and R2729S may thus not exhibit an arrhythmia phenotype. In the mouse, it was proposed that the mutation alters RyR2 structure such that the whole complex becomes more susceptible to protein kinase A (PKA)-mediated phosphorylation modulation, thus causing increased arrhythmia. This could occur either by increased propensity to ‘unzipping’ of the N-terminal and central domains, and thus result in a significant increase in the frequency of spontaneous Ca^2+^ transients as shown in R2474S/ + knock-in CM^28^. Alternatively, it could be effected by dissociation of FKBP12.6 from human RyR2 following PKA phosphorylation^65^. In *C. elegans*, no adrenergic signaling is present; however, serotonin and other neuromodulators affect pharynx function as a response to the presence or absence of food. Yet, food-stimulated pumping was unaffected in the R2729S mutant animal (Fig. 2e). Last, we analyzed a homozygous mutant, while in mouse studies, heterozygotes were used, which may underlie discrepancies in the two systems.

The second CPVT-related mutation R4743C (R4497C in humans), as well as the deletion of *unc-68*, caused a significant progressive decrease in the ability to follow paced pumping at high rate, both in EPG-recordings and video microscopy of intact animals, where the majority of animals were able to follow only every second light pulse or stopped pumping. This finding manifests a ‘worm arrhythmia’ phenotype of the CPVT-like mutation in our optogenetic model. The equivalent mutation R4496C in mice was suggested to play an essential role in RyR2 channel gating, and it was shown that the channel is more likely to open even at low Ca^2+^ concentration^30^. Thus, a faster depletion of Ca^2+^ could be underlying the inability of UNC-68(R4743C) pharynxes to follow 3.7 Hz pacing.

The arrhythmic phenotype observed in the pharynx of the *C. elegans* mutant UNC-68(R4743C) argues for our system as a valuable arrhythmia model that enables to study CPVT mutations. To assess whether this also enables drug testing, we analyzed effects of the benzothiazepine S107 on restoring the pump ability of the arrhythmic pharynx. Indeed this was possible. It is interesting that S107 was considered to stabilize the FKBP12.6-RyR2 interaction in humans, yet, here it also showed beneficial effects for R4743C (R4497C in humans). This strengthens the assumption that the respective mutation influences the FKBP12.6-RyR2 interaction^65^, as already shown by Lehnart *et al*.^25^ when using S107 treatment for the R2474S mutation. Our work demonstrates that potential anti-arrhythmogenic drugs can be studied in the *C. elegans* system, which may be used as a rapid, initial model for introducing patient-specific CPVT mutations, and then to screen potential drugs in a relatively straightforward manner. Of course, this may not always be possible if the affected residue in humans is not conserved in UNC-68. However, possibly the human RyR2 can be introduced into *C. elegans* to replace UNC-68, thus generating a ‘humanized’ model. Worms can be treated with drugs and then assessed in a high-throughput manner by video microscopy.

In conclusion, we have established the pharynx of *C. elegans* as a model to characterize CPVT mutations, for two loci affected by this genetic condition, CASQ2 and RyR2 (*csq-1* and *unc-68* in *C. elegans*, respectively). The optogenetically paced pharynx model enables testing of drugs like S107 on specific mutations. The approach may enable characterizing new CPVT mutations found in patients, but possibly also mutations of RyR1, which are associated with malignant hyperthermia or central core disease.

## Methods

### *C. elegans* strains

*C. elegans* strains were cultivated at 20 °C on nematode growth medium (NGM) fed with *E. coli* strain OP-50-1. We used the following strains kindly provided by the *Caenorhabditis* Genetics Center (CGC): N2 (wild type), **RB2019**: *csq-1(ok2672)X*, **VC40907:** *csq-1(gk876502)X*; altering P319 to S, **TR2171:** *unc-68(r1162)V*, **CB540:** *unc-68(e540)V*, **JT73**: *itr-1(sa73)IV* and **RB2222**: *fkb-2(ok3007)I. unc-13(s69)I* was kindly provided by E. Jorgensen.

Furthermore, we generated the following transgenic strains: **ZX1534:** *csq-1(ok2672)X; zxEx754[pcsq-1(2.4 kb)::csq-1::CFP (5ng); pmyo-3::mCherry]; zxIs20[pmyo-2::ChR2(H134R)::mCherry; pges-1::nls::GFP]*, **ZX1581:** *csq-1(ok2672)X; zxEx754[pcsq-1(2.4 kb)::csq-1::CFP (5ng); pmyo-3::mCherry]*, **ZX1651:** *unc-68(r1162)V; zxEx752[punc-68::unc-68 (1ng); pmyo-3::mCherry]; zxIs20 [pmyo2::ChR2(H134R)::mCherry; pges-1::nls::GFP]*, **ZX1654:** *unc-68(r1162)V; zxEx749[punc-68::unc-68(R2729S) (1ng); pelt-2::mCherry)]; zxIs20[pmyo2::ChR2(H134R)::mCherry; pges-1::nls::GFP]*, **ZX1819:** *csq-1(gk876502)X; zxIs20[pmyo-2::ChR2(H134R)::mCherry; pges-1::nls::GFP]*, **ZX1833:** *unc-68(r1162)V; zxEx748[punc-68::unc-68(R4743C) (1ng), pelt-2::mCherry]; zxIs20[pmyo2::ChR2(H134R)::mCherry; pges-1::nls::GFP]*, **ZX2121:** *unc-68(r1162)V; punc-68::unc-68 (exon1–4)::CFP (10 ng* *)*.

### Plasmids

Plasmid *pmyo-2::ChR2(H134R)::mCherry* was generated as described in Schuler *et al*.^21^.

The *csq-1* gene was amplified by PCR from *C. elegans* genomic DNA (primer pair: oEF48, oEF49) and inserted into *pmyo-3::CFP* (based on pPD115.46) via restriction sites BamHI and KpnI. All primer sequences are listed in Supplementary Table S1. The *myo-3* promoter was exchanged to the endogenous *csq-1* promoter, by obtaining a 2.4 kb DNA fragment upstream of the CSQ-1 encoding region by PCR from the genomic DNA (octs3, octs4) and insertion into the *pmyo-3::csq-1::CFP* plasmid via PstI and BsaBI restriction sites, resulting in *pcsq-1::csq-1::CFP*.

*Punc-68::unc-68(exon1–4)::CFP*, which was used for the verification of expression via *punc-68*, was generated using the In-Fusion cloning HD Kit (Clontec Takara Bio Europe, Saint-Germain-en-Laye, France). The *pmyo-2::CFP* plasmid was restricted by Acc65I and SalI and served as backbone. *punc-68::unc-68* was amplified from fosmid WRM063aG12 by primer pair oEF150 and oEF151 containing homology arms annealing to the restricted *pmyo-2::CFP* plasmid and ligated.

### Generation of *unc-68* point mutations

Insertion of point mutations in the *unc-68* gene was performed by a modified protocol of the heat induced recombination strategy in fosmids previously published by Tursun *et al*.^66^. Electro-competent *E. coli* SW105 which contain an arabinose-activatable FLP recombinase (not used in these experiments) and a heat-activatable λ red recombinase, were transfected with fosmid WRM063aG12 by electroporation (MicroPulser™ Electroporator, Biorad, California, USA) at 2000 V, 6 ms. Successful transfection was verified by isolation of fosmid DNA and test digestion with AgeI. For insertion of point mutations, a two-step recombination and selection strategy was used. For the first step, λ red recombinase was heat-activated and electro-competent SW105 containing fosmid WRM063aG12 were generated^66^ and transfected with a PCR product containing a galactokinase-K (GalK) encoding gene with corresponding em-7 promoter, serving as a selection marker and flanked by 50 bp *unc-68*-homology sequences including the respective point mutation (Supplementary Fig. 4, Step 1). The cassette was amplified from the pBALU-1 plasmid^66^, with primers containing 50 bp of *unc-68* homology (R2729S: oEF200, oEF201; R4743C: oEF202, oEF203).

Recombined clones were selected on galactose minimal agar plates and analyzed by colony-polymerase chain reaction (PCR; R4743C: oEF15, oEF16; R2729S: oEF204, oEF205). Mixed clones containing both WT-*unc-68* and *GalK*-*unc-68* fosmids were observed and “only*GalK*” clones were separated by repeatedly streaking out on MacConkey agar plates^67^, containing the pH indicator neutral red for an indication of galactose degradation, and analyzed via colony PCR screening (Supplementary Fig. 4, Step 1). The *GalK* sequence was replaced by the respective point mutation in a second step as follows: Mutation-specific PCR products, containing ~350 bp of homology and the point mutation were used for the second λ red recombinase and selection step (Supplementary Fig. 4, Step 2). Using fosmid WRM063aG12 as template these PCR products were generated by overlap PCR. At first PCR fragments containing the point mutations and overlapping regions were amplified with oEF206, oEF207 and oEF208, oEF209 for the R2729S mutation as well as oEF210, oEF211 and oEF212, oEF213 for the R4743C mutation, verified by gel electrophoresis and purified by gel extraction. In a second PCR reaction, the fragments were combined by overlap PCR to a final recombineering construct with the mutant corresponding primer pairs oEF206, oEF209 for R2729S and oEF210, oEF213 for R4743C. Electro competent, λ red recombinase heat-activated SW105-“only*GalK*” bacteria were generated, transfected with the corresponding PCR product and cells were selected on deoxy-galactose minimal agar plates (Supplementary Fig. 4, Step 2). Colony-selection occurred via colony PCR with primers and the final *unc-68* constructs with the desired point mutations were verified by sequencing of colony PCR products with primers (R4743C: oEF15, oEF16; R2729S: oEF204, oEF205) (Supplementary Fig. 4, Step 2).

### Microinjection and generation of transgenic animals

Transgenic animals expressing endogenous UNC-68 or UNC-68 with point mutations, respectively, were generated by microinjection of 1 ng/µl of the particular fosmid and the co-injection marker *pmyo-3::mCherry* (10 ng/µl) or *pelt-2::mCherry* (20 ng/µl) in the deletion mutant *unc-68(r1162)*. Generation of a strain for verification of *unc-68* expression in the pharynx occurred by microinjection of 10 ng/µl of *punc-68::unc-68(exon1–4)::CFP* in the deletion background *unc-68(r1162)V*. The CSQ-1 rescue construct was expressed in mutant *csq-1(ok2672)* after microinjection of 5ng/µl *pcsq-1(2.4 kb)::csq-1::CFP* with co-injection marker *pmyo-3::mCherry* (10 ng/µl).

### Genotyping of mutants

*unc-68(r1162)V* mutants were selected by locomotion phenotype, and were confirmed by PCR genotyping (oEF59, oEF60, oEF61). Genotyping of *csq-1(ok2672)X* was likewise done by PCR (oNH1, oNH2, oNH3). The mutation *csq-1(P319S)* ^68^ was genotyped by PCR amplification (oEF214, oEF215), digestion with HinfI and analysis by electrophoresis on a 5% acrylamide gel to visualize the resulting small DNA fragments. The positively genotyped mutants were verified by sequencing.

### Fluorescence microscopy

Expression of *pcsq-1::csq-1::CFP* and *punc-68::unc-68(exon1–4)::CFP* was analyzed on a Zeiss Axio Observer microscope, with an 40x/0.25 Zeiss ∞/- APlan oil objective and CFP filter set (Carl Zeiss, Göttingen, Germany). Animals were transferred on 2% agarose pads in M9 buffer (K_2_PO_4_, 20 mM; Na_2_HPO_4_, 40 mM; NaCl, 80 mM; MgSO_4_, 1 mM) and immobilized with 1 µL freshly prepared 50 mM sodium azide solution (Sigma-Aldrich, USA, St. Louis) in M9 buffer from a 1 M stock in water.

### Determination of spontaneous pump rate on food

One day before experiments, L4 larvae were picked on NGM dishes (55 mm, 8 ml NGM) seeded with 320 µl OP50 culture. Spontaneous pumping of animals on food was video recorded (Powershot G9, Canon, Tokio, Japan) and visually counted for 20 s. Mean values, standard error of the mean (SEM) and further statistics (1-way ANOVA with Bonferroni post-hoc test) were calculated with OriginPro (OriginLab, Northampton, MA, USA).

### EPG-Recording and optical pacing

One day before experiments young adult hermaphrodites were placed on fresh NGM plates with or without all-*trans* retinal (ATR, Sigma Aldrich, St. Louis, MO, USA). ATR (0.65 µl of a 100 mM stock in ethanol) was added to 650 µl of OP50 culture and spread onto 94 mm culture dishes (vented, Greiner Bio-One, Kremsmuenster, Austria) containing 25.2 ml of NGM. We performed EPG recordings on intact animals and on cut head preparations. For cut head preparations, animals were transferred into a recording chamber containing a Sylgard-coated cover slip (25 mm diameter) and filled with 1.5 ml of EmD50 buffer (NaCl, 140 mM; KCl, 3 mM; CaCl_2_, 3 mM; MgCl_2_, 1 mM; Hepes, 10 mM; D-Mannitol, 50 mM; pH 7.3 adjusted with NaOH). The head of an animal was cut away from the body with a scalpel (Braun Aesculap, Tuttlingen, Germany) directly posterior to the terminal bulb. Upon dissection, the body wall muscles contract and expose the posterior pharynx.

Electrophysiology was performed as described in Schuler *et al*.^21^. The tip of the worm head was sucked into an EPG-suction electrode (~20 µm inner diameter tip) using a 10x objective. For optical pacing with a 470 nm LED (KSL-70, Rapp Optoelectronics, Hamburg, Germany) the pharynx was positioned below a 60x water immersion objective (LUMIplan FI/IR, 0.9 NA); an EGFP-ET filter set (AHF Analysentechnik AG, Tuebingen, Germany) was used. Recording of EPGs and triggering of light pulses was synchronized by PatchMaster software (Heka, Lambrecht, Germany).

Either spontaneous pumping (at least for 1 minute) was recorded or the pharynx was stimulated with 470 nm light pulses (1.5–2 mW/mm², 35 ms) over a period of 30 s or in a stress test (stepwise increasing stimulation frequency: 1–7 Hz; pulse duration: 10 ms, over a period of 5 seconds each). Review software (Bruxton Corporation, Seattle, WA, USA) was used to translate PatchMaster files to ABF files. Pump rate and duration were analyzed by AutoEPG^53^ (kindly provided by Dr. Christopher James, Embody Biosignals Ltd., UK). Excel was used for calculation of means and SEM, 1-way ANOVA with Bonferroni post-hoc test was performed using OriginPro.

### High-throughput kymograph recording and optical pacing

Transgenic L4 larvae cultivated with ATR were placed on fresh NGM dishes containing ATR one day prior to the assay. 1 µL of polystyrene microspheres (Polysciences 00876-15, 2.5% w/v suspension, Hirschberg an der Bergstrasse, Germany) were added on pads composed of 10% agarose. About 10 animals were transferred into the beads and gently overlaid with a coverslip.

Measurements were performed on a Zeiss Axio Observer, equipped with a 100 W HBO lamp, EGFP Filter (Ex. 472/30, beam splitter 570, Em. 675/50) and an 10x/0.25 Zeiss ∞/- APlan objective. Animals were stimulated with blue light pulses (35 ms, 1.5 mW/mm²) with a computer-controlled shutter (Sutter Instruments, Novato, CA, USA) every 250 ms (4 Hz) over a period of 30 s. Recording was performed using an ORCA Flash 4.0 sCMOS camera (Hamamatsu, Japan; 20 fps, 10 ms exposure time, 2 × 2 binning, 1024 × 1024 Pixel) and µManager v1.4 software. For drug tests, animals were incubated 30 min in 50 µL of 50 µM S107 (Calbiochem-Order number: 500469; Merck, Darmstadt, Germany) in M9 buffer containing 0.1% DMSO before the measurements.

Multi-kymographs of grinder movements were generated and analyzed as described in Schuler *et al*.^21^. Excel was used for calculation of means and SEM, 1-way ANOVA with Bonferroni post-hoc test was performed using OriginPro.

### Data availability

The data that support the findings of this study (video recordings, original records of electrophysiological data) are available from the corresponding authors upon reasonable request.

## Electronic supplementary material

Supplements
