# Supplementary material for: An optogenetic arrhythmia model to study catecholaminergic polymorphic ventricular tachycardia mutations
Source: Sci Rep. 2017 Dec 13;7:17514. doi: 10.1038/s41598-017-17819-8 (PMC5727474; doi:10.1038/s41598-017-17819-8)
Supplement: Supplementary file 1 — Supplements [file 41598_2017_17819_MOESM1_ESM.pdf]

An optogenetic arrhythmia model to study catecholaminergic polymorphic ventricular tachycardia mutations

Elisabeth Fischer[1,2,4] , Alexander Gottschalk[1,2,3,*] & Christina Schüler[1,2,*]

[1]Buchmann Institute for Molecular Life Sciences, Goethe University, Max von Laue Strasse 15, D-60438 Frankfurt, Germany

[2]Institute of Biophysical Chemistry, Goethe University, Max von Laue Strasse 15, D-60438 Frankfurt, Germany

[3]Cluster of Excellence Frankfurt – Macromolecular Complexes, Goethe University, Max von Laue Strasse 15, D-60438 Frankfurt, Germany

[4] Present address: University of Edinburgh, Centre for Integrative Physiology, Hugh Robson Building, George Square, Edinburgh, EH8 9XE, UK

[*]to whom correspondence should be addressed: a.gottschalk@em.uni-frankfurt.de & c.schueler@em.uni-frankfurt.de

Supplementary Material

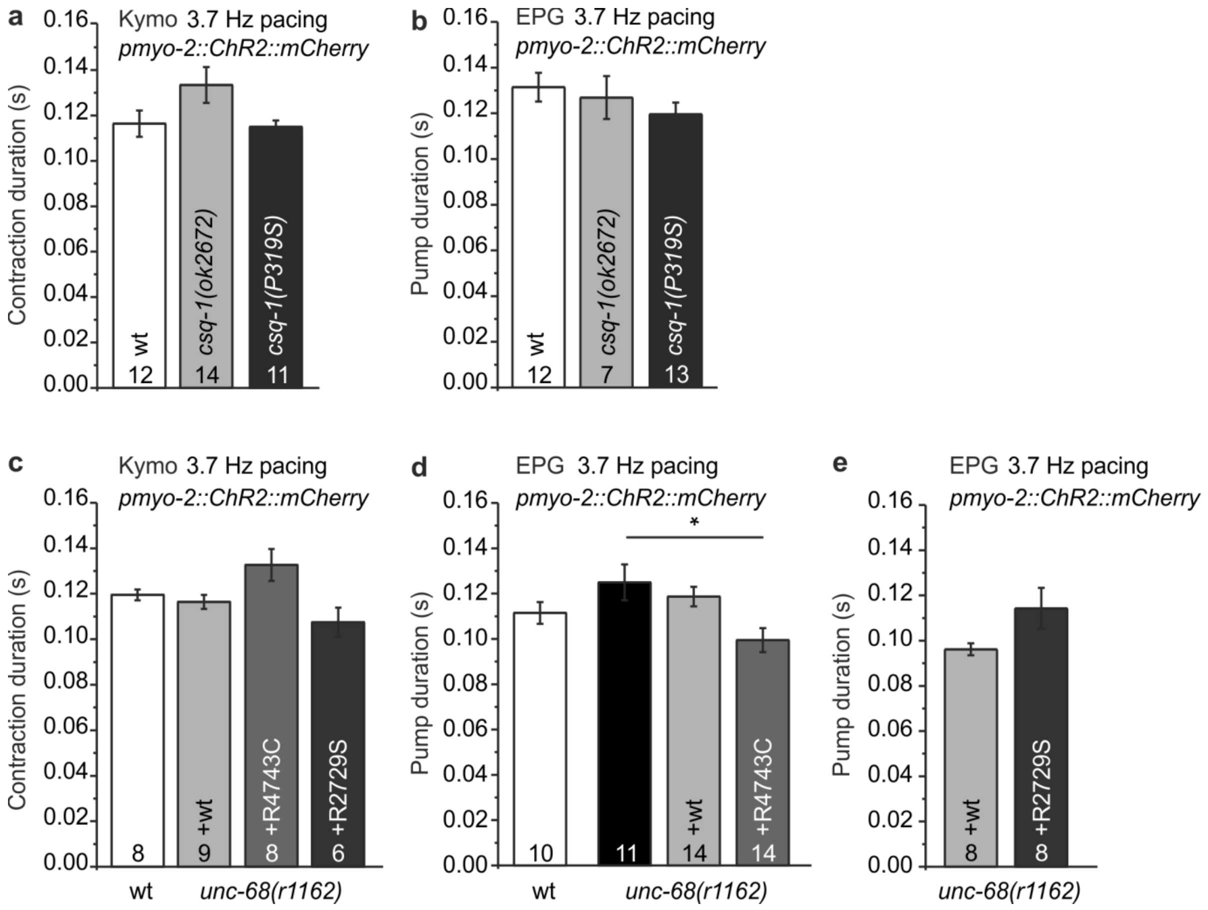

Supplementary Figure 1│ Mutations of *unc-68* and *csq-1* do not affect pharynx pump duration. (**a**) Contraction duration obtained by kymographic video analysis of intact animals and (**b**) pump duration obtained by EPG recordings of cut-head preparations of deletion mutant *csq-1(ok2672)X.* and point mutation *csq-1*(*gk876502*) (altering P319 to S), compared to wt (n = 11-14, n=7-13, respectively). (**c**) Contraction duration obtained by kymographic video analysis and (**d, e**) pump duration obtained by EPG recordings of intact animals of *unc-68(r1162)* deletion mutant, its rescue with *unc-68* wt fosmid as well as the R4743C and R2729S point mutant fosmids, compared to wt (n=6-12, n=10-14, n=8, respectively).

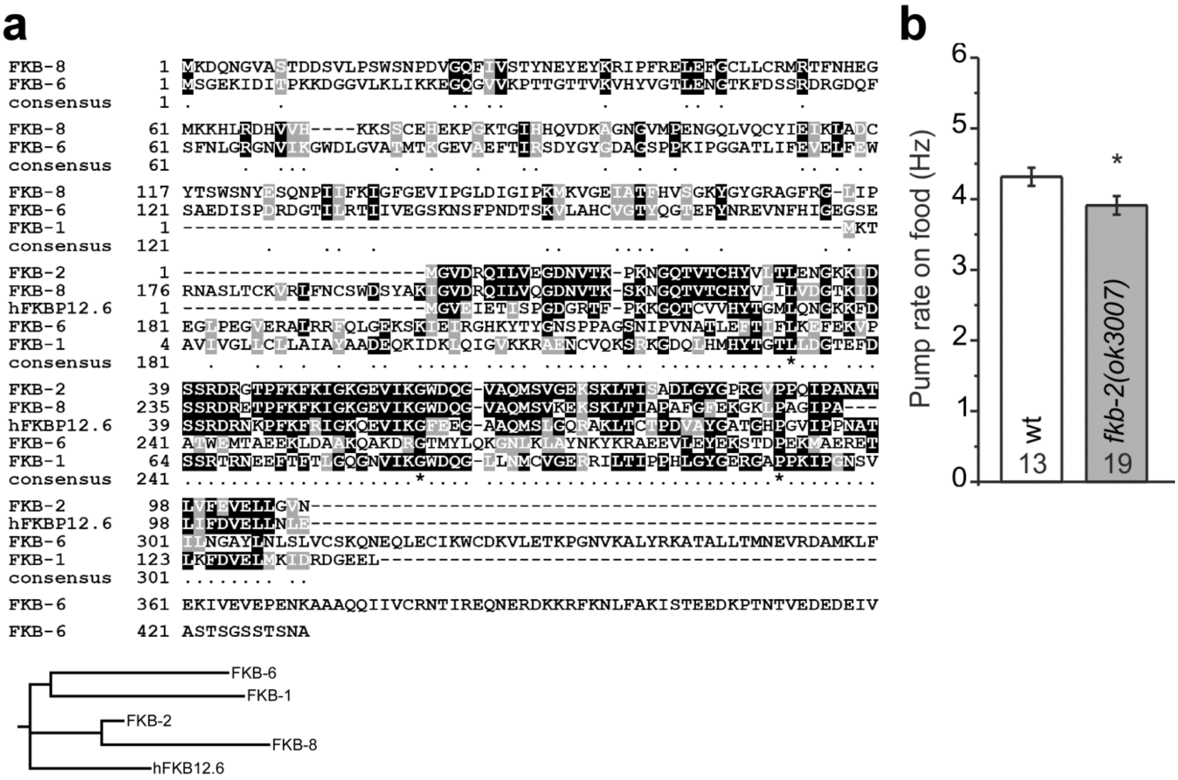

Supplementary Figure 2│ Deletion of FKB-2, the presumptive *C. elegans* homolog of human FKBP12.6, reduces pump rate on food. (a) Clustal-X2 alignment of *C. elegans* sequences of FKB-1, FKB-2, FKB-6 and FKB-8, with human FKBP12.6. Inset: Phylogenetic tree of the five proteins. (**b**) Food-stimulated pumping was compared in wt and *fkb-2(ok3007)* deletion mutants, by video microscopy.

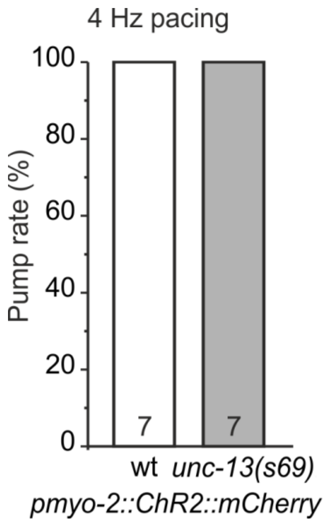

Supplementary Figure 3│ No neuronal influence on paced pharynx pumping. EPG recordings of cut-head preparations of *pmyo-2::ChR2::mCherry*-expressing animals containing the *unc-13(s69)I.* mutation compared to wt (n =7).

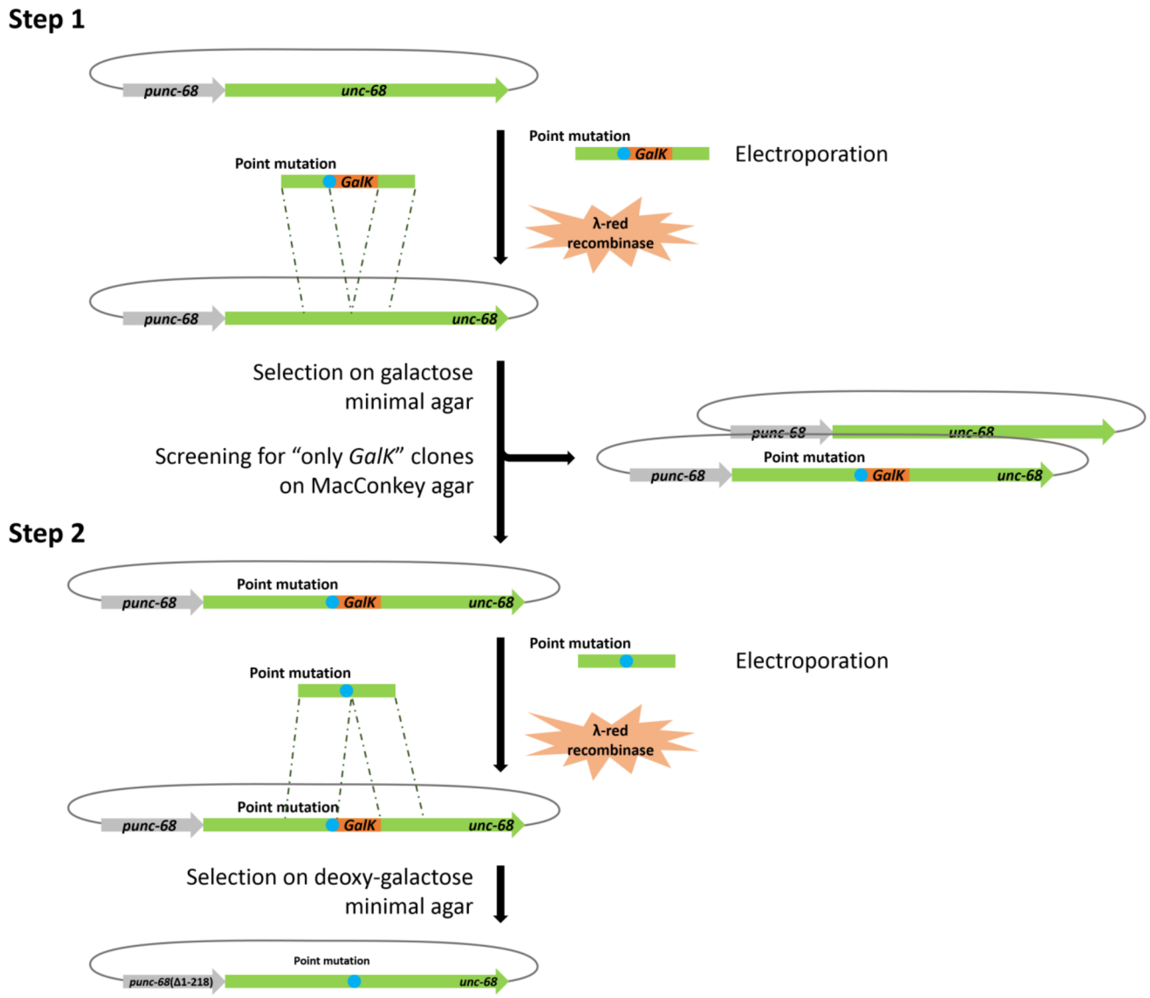

Supplementary Figure S4│ Heterologous recombineering. Insertion of point mutations into a fosmid containing *unc-68* by heterologous recombineering using SW105 cells, the recombinase λ-Red and GalK cassette for selection (see Methods for details).

Supplementary Table 1: Primer sequences

Primers for generation of plasmids used in this work:

oEF48: 5`-GCTCGGATCCATGACTTGGATCAAACTTGC-3`

oEF49: 5`-GCTGGGTACCGGCAGCTCCTTTCTAGACTTCTTTCC-3`

octs3: 5`-CATCGCTGCAGGTTACAGGCATCGATTG-3`

octs4: 5`-GTTCTTCTCGGTCAGAAC-3`

oEF150: 5`-ATGCCTGCAGGTCGAAGCCTTTCGGCGCGACTAATA-3`

oEF151: 5`-TTTTTCTACCGGTACTAACAGTGTTTTGTGACCAC-3`

Primers used in this work for generation of *unc-68* point mutations in fosmids:

oEF200: 5`-TACCAGGCCTACTTCCAAACCATAAGGGATCAGTTTTGCTGTTCTTAGATTCACCTGTTGA CAATTAATCATCGG-`3

oEF201: 5`-GATTGCTCAAGAACATGGAAGAGCATATCTTGTTGATCAATTCCGTATACGCGGCCGCTC TAGAACTAGTGGATCC-3`

oEF202: 5`-TACAGATGTACAAAAACTCAATATTTAAATATTTCCAGAATATGCTTGCATGTCCTGTTGA CAATT AATCATCGG-3`

oEF203: 5`-TTGATGAAGAAAGCCAAGTACAAAGTGATCTTTTCGATTGTTTTGAAGTTGCGGCCGCTC TAGAACTAGTGGATCC-3`

oEF15: 5`-CTTCATCGTCGTCTCCTTCC-3`

oEF16: 5`-GTGCACCTCAAATGGTTCAG-3`

oEF204: 5`-GAAGATCCAGCCGATTCAAC-3`

oEF205: 5`-TTGAGCGCAAGAGCTGTATC-3`

oEF206: 5`-AAGATCCAGCCGATTCAACC-3`

oEF207: 5`-CAATTCCGTATACTGAATCTAAGAACAGC-3`

oEF208: 5`-GCTGTTCTTAGATTCAGTATACGGAATTG-3`

oEF209: 5`-CGTCGGCAAAGAAGTGTGAG-3`

oEF210: 5`-GAATCTATCGGTGCACCTC-3`

oEF211: 5`-CCAGAATATGCTTGCATGTAACTTCAAAACAATCG-3`

oEF212: 5`-CGATTGTTTTGAAGTTACATGCAAGCATATTCTGG-3`

oEF213: 5`-CCTTCCGATGCAGCTTCAGC-3`

Primers used in this work for genotyping of mutants:

oEF59:	5`-CTCAGCGGTGGAAAC-3`
oEF60:	5`-GTACTTGAACCTAGGTAC-3`
oEF61:	5`-GTGTACTCTGTTTCGTC-3`
oNH1:	5`-TGCGCAGACCATTTGAGGAAG-3`
oNH2:	5`-CTTCCCTCACGGGTTTCCTC-3`
oNH3:	5`-GAAACTACCAAGCGGCAACC-3`
oEF214:	5`-TGCGCAGACCATTTGAGGAA-3`
oEF215:	5`-GAAGGACCTCAAATCGGACT-3`
